# Coating Techniques for Functional Enhancement of Metal Implants for Bone Replacement: A Review

**DOI:** 10.3390/ma12111795

**Published:** 2019-06-03

**Authors:** Amir Dehghanghadikolaei, Behzad Fotovvati

**Affiliations:** 1 School of Mechanical, Industrial and Manufacturing Engineering, Oregon State University, Corvallis, OR 97331, USA; dehghana@oregonstate.edu; 2 Department of Mechanical Engineering, The University of Memphis, Memphis, TN 38152, USA

**Keywords:** surface modification, biocompatible metals, coating techniques, hydroxyapatite

## Abstract

To facilitate patient healing in injuries and bone fractures, metallic implants have been in use for a long time. As metallic biomaterials have offered desirable mechanical strength higher than the stiffness of human bone, they have maintained their place. However, in many case studies, it has been observed that these metallic biomaterials undergo a series of corrosion reactions in human body fluid. The products of these reactions are released metallic ions, which are toxic in high dosages. On the other hand, as these metallic implants have different material structures and compositions than that of human bone, the process of healing takes a longer time and bone/implant interface forms slower. To resolve this issue, researchers have proposed depositing coatings, such as hydroxyapatite (HA), polycaprolactone (PCL), metallic oxides (e.g., TiO_2_, Al_2_O_3_), etc., on implant substrates in order to enhance bone/implant interaction while covering the substrate from corrosion. Due to many useful HA characteristics, the outcome of various studies has proved that after coating with HA, the implants enjoy enhanced corrosion resistance and less metallic ion release while the bone ingrowth has been increased. As a result, a significant reduction in patient healing time with less loss of mechanical strength of implants has been achieved. Some of the most reliable coating processes for biomaterials, to date, capable of depositing HA on implant substrate are known as sol-gel, high-velocity oxy-fuel-based deposition, plasma spraying, and electrochemical coatings. In this article, all these coating methods are categorized and investigated, and a comparative study of these techniques is presented.

## 1. Introduction

Metallic biomaterials have been mostly used for body implants thanks to their various properties, such as mechanical strength, corrosion resistance, and biocompatibility. Although there are several metallic elements and alloys, a few of them (such as Titanium (Ti), Ti-based alloys, Platinum (Pt), and austenitic stainless steel (316L)) are implemented for orthopedic and biomedical applications [1,2,3]. However, due to the nature of metal-corrosive media interaction, degradation takes place after the implementation of these materials inside the human body. Some of the products of the corrosion reactions are harmful to the living organs adjacent to the implants. Nickel (Ni) ions released from corrosion of nickel–titanium (NiTi) alloy implants are one of the examples of these byproducts [4,5]. One of the most important aspects of the suitability of a material for bio-applications is to have higher corrosion resistance and, consequently, lower toxicity due to released metallic ions [6]. Any difference between the chemical composition of the bone structure and the metallic implants causes bone/implant bonding issues and subsequent problems for a patient [7,8]. To solve the corrosion and bone/implant bonding issues, many researchers have suggested surface treatment by bioactive hydroxyapatite (HA) ceramic coating. This coating consists of Ca/P components (Ca_10_(PO_4_)_6_OH_2_), enhances the bone/implant bonding properties, and increases the corrosion resistance of the substrate [9,10]. Corrosion measurements are mostly done in prepared simulated body fluids (SBF) and one of the most common types of SBF is Hank’s solution which is mostly consisted of NaCl [11]. HA coatings have a close composition match with that of the bones as the components are the major inorganic portion of the bone composition. Thanks to this match, these coatings allow fast and selective bone ingrowth and enhanced osseointegration [12,13].

Corrosion resistance, biocompatibility, wear resistance, stiffness match with bone, and enhanced bone ingrowth are the most effective features of bone implant materials [14,15,16]. Corrosion resistance and biocompatibility are the most crucial indicators of suitability of metallic implants while exposed to harsh environments. Human body environment and physiological fluids are corrosive saline mediums which cause noticeable corrosion of implants if they are not protected by oxide layers or protective covers. In some of the metallic materials which have specific elements such as nickel (Ni), the byproducts of the corrosion reactions happening inside the human body can be severely toxic and lethal to their adjacent living tissues. Concurrently, the corrosion process degrades the implant materials and reduces their mechanical stability which eventually causes a premature failure (before complete healing of the patient). Even if none of the above effects harm the patient, a secondary surgery is needed in order to remove the implant out of the patient body after complete recovery [17,18].

Other than corrosion resistance, biocompatibility is the most important functional feature of implant materials. Biocompatibility is defined as the reaction of living organs to the implant material around them. If the tissues present positive feedback and live in contact with the implants, the material is biocompatible, and cells can grow and sustain on or close to the implant surface. However, if there are toxic ions released from the implant material, cell growth will be prevented. This characteristic relies on the substrate microstructure and chemical composition as well as the quality of the surface of the material such as surface roughness, which depends on the manufacturing processes such as machining [19,20,21,22,23].

All being said, the coating processes and especially the ones offering deposition of HA, metallic oxides, and polymers are reliable solutions. In the following discussion, the most applicable techniques are introduced in detail, i.e., sol-gel, high-velocity oxy-fuel plasma coating (HVOF), plasma spraying, and electrochemical coatings. Although the mentioned processes are widely used in the deposition of protective layers and surface treatment, there are other processes which are more advanced and are mostly used for specific applications. Instances of these processes are laser beam melting (LBM) electron beam melting (EBM), and ion beam melting (IBM) processes that utilize the emitted energy of the electron, ion, and laser beams to melt materials and deposit the melt on a substrate surface. In addition, these processes are known as high-energy coating techniques which are less used for common applications [24,25,26,27,28,29]. The coating quality in these methods is affected by the melt pool characteristics, which depend on the process parameters [30]. Moreover, different modeling and simulation techniques can be implemented to achieve a deeper understanding of the deposition processes. These methods can utilize continuum mechanics principles, numerical solutions, and use of software to release the highest possible accuracy in their predictions [31,32,33,34].

In summary, this study intends to introduce the most efficient means of surface protection and coating for biomedical materials, especially the ones used as bone implants, and evaluate their potential as a reliable way to deposit the desired materials on the surface of implants with the least possible side-effects. The following sections are talking about the coating techniques and the deposited layers with the most significant advantage they provide.

## 2. Bioactive Material Deposition Techniques

One of the most common surface modification processes is the deposition of a set of selected materials, called coating. However, since the range of these materials is wide, different methods have been introduced. These processes are selected based on the substrate material, applications of the coated material, and the coating layer thickness [35,36]. There are numerous coating methods offering different capabilities each, however, only a few of these techniques are sufficiently reliable to be applied for bio-application purposes [37,38]. These techniques simultaneously provide corrosion resistance and biocompatibility enhancement for the substrate. Among many materials for these means, using HA shows a high increase in biocompatibility and bone/implant interface formation. The following discussion presents reliable methods (i.e., sol-gel technique, High-Velocity Suspension Flame-Spraying (HVSFS), plasma spray coating, and electrochemical deposition) of HA deposition [39,40].

### 2.1. Sol-gel

**Figure 1:**
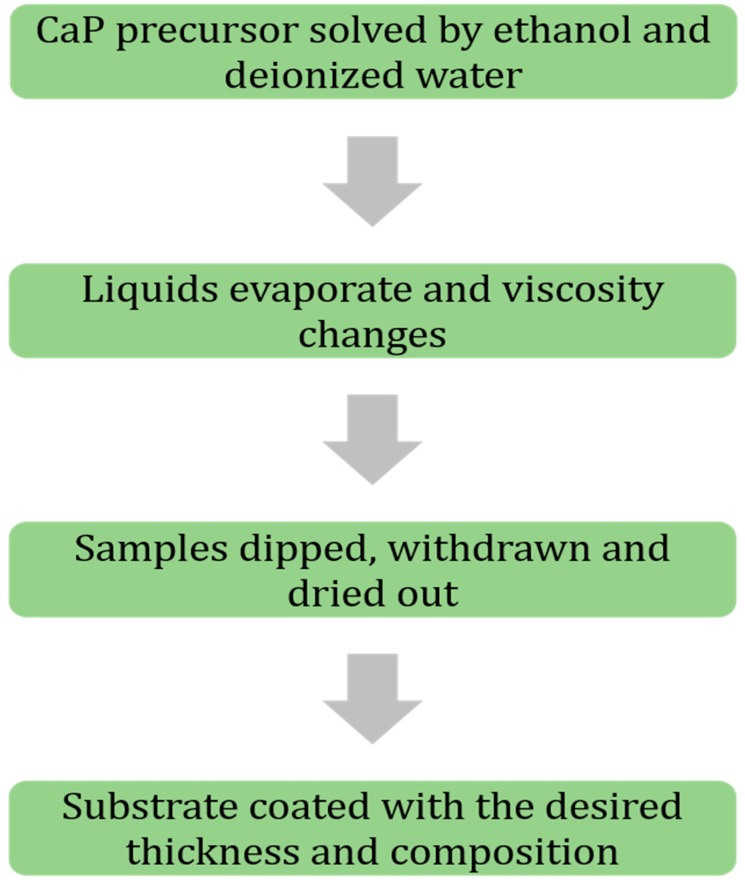
Flowchart of sol-gel coating process coating steps, in brief.

Sol-gel has been at the center of attention in recent years thanks to its simplicity, flexibility, and low cost of the process. This process provides a reliable enhancement of coating adhesion on the substrate of metallic biomaterials [35,41]. Sol-gel consists of two distinct parts known as sol and gel. For the sol part, calcium-phosphorus-based (CaP) precursors are solved in ethanol and distilled water to produce phosphorus pentoxide (P_2_O_5_)/triethyl phosphate (C_6_H_15_O_4_P) and increase hydrolysis of the sol part, respectively [42,43,44]. Ethanol plays a key role in solving Ca part of the precursors, as well [45]. In the next step, the two distinct parts are mixed carefully, and after undergoing an evaporation process, the liquid medium goes away. This process repeats for many times until the desired viscosity of the sol-gel medium is achieved. Besides the viscosity, the chemical concentration of the ingredients is crucial in achieving a high HA formation on substrates [46,47]. The sol-gel technique is a dipping process that undergoes three different steps: Dipping, withdrawing, and air drying. The first two steps are mostly done in a controlled constant speed to prevent entrapping air bubbles and a non-uniform layer thickness of the coating medium, respectively. To obtain such a quality of uniformity, many researchers utilize controlled speed motors or servo motors. However, there has not been a significant difference between parts coated manually and automatically since the process is a dipping method and covers the whole geometry of the substrate regardless of its complexity [48,49,50]. On the other hand, this process can perform composite and multi-layer coatings by changing sol-gel medium (i.e., composition and viscosity) and iteration of dipping, withdrawing, and drying steps, respectively. With this method, it has been reported that HA coating of 0.05–15 mm thickness has been achieved [35,51]. Based on drying and the method of applying a sol-gel coating (dipping and rotation) to the substrate, different structure of coating (rods and spheres) can be achieved [35]. Figure 1 represents a summary of the steps of the sol-gel coating process.

**Figure 2:**
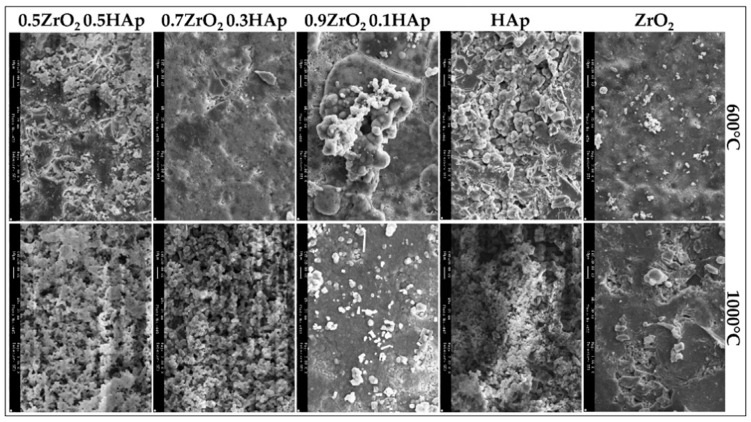
SEM micrographs of composite coated samples heated to 600 °C and 1000 °C after 21 days of exposure to simulated body fluids (SBF) with different compositions [62].

Another advantage of sol-gel coatings can be the ability to undergo annealing in a furnace to further stabilize the deposited layers with less deep thermal cracks, which cause discontinuity and less protection [52]. Compared to the thermal coating processes, there is no significant change in the composition of the deposited layer of coating. Kuntin et al. [53], reported that deposited HA layer on the substrate by plasma spraying decomposes to calcium oxide (CaO), tetra-calcium phosphate, and tricalcium phosphate. The high temperatures of these types of coating processes (above 5000 °C) may burn or delaminate the deposited layers, as well. However, annealing temperatures used for curing and stabilization of sol-gel deposited layers are in the range of 375 to 500 °C that is notably lower than those of thermal coatings [54]. Liu et al. [55], reported a significant adhesion enhancement of HA layers after annealing in an atmospheric protected furnace in the mentioned temperature range. In other scientific reports, it has been mentioned that post-processes such as heat treatment facilitates densification and apatite formation of the deposited HA materials on the substrates and increases adhesion between the substrate and the deposited materials. However, the heat treatment temperatures must be below the melting point of the weakest material in order not to force any collapse in the mechanical and biological properties [7,56,57]. Other considerations have been applied to improve the quality of sol-gel deposited HA layers. For instance, a new polymeric material known as poly ε-caprolactone (PCL) has been introduced [58]. Hanas et al. [59], reported the formation of a porous microstructure of the coating layer and increased osseointegration of coated substrates by addition of PCL to HA. Alemon et al. [60], claimed that a porous coating layer of 184 μm thickness was formed on a Ti6Al4V substrate that provided enhanced adhesion between coating and substrate with fewer micro-cracks on the surface of the coating layer. In a similar study by Catauro et al. [61], they reported an increased wear and corrosion resistance of the coated substrates resulting in less metallic ion release as corrosion byproducts. Figure 2 shows a composite sol-gel coating microstructure. In this figure, the sol-gel coated samples were post-processed in 600 and 1000 °C and then were exposed to SBF media for 21 days. The difference in morphology of the coating surface can be clearly seen for different heat treatment temperatures and medium compositions.

### 2.2. High-Velocity Suspension Flame-Spray (HVSFS) Coating

**Figure 3:**
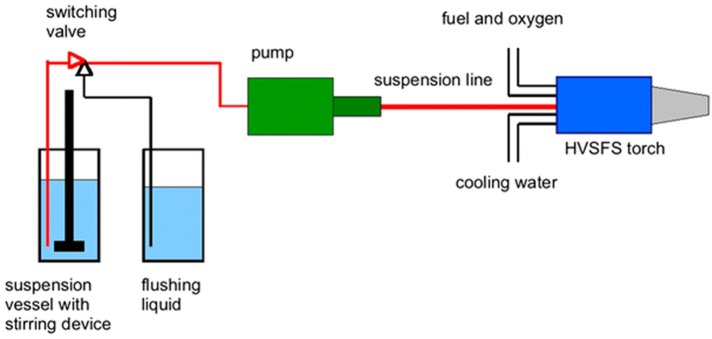
Schematic illustration of a high-velocity suspension flame-spray (HVSFS) experimental setup for nano-oxide ceramic coatings [73].

HVSFS is a modified type of high-velocity oxy-fuel (HVOF) coating process that utilizes suspensions with the desired composition to deposit a coating layer on substrates [63,64]. Figure 3 represents a schematic view of an experimental HVSFS setup. In this method, an inlet pushes the coating materials to the stream of hot flames and accelerates them toward the target substrate. The advantage of using this process is a significantly increased coating speed and a larger area of coverage [65,66]. The principles of this process are straightforward, and the equipment are not expensive compared to other advanced coating technologies such as EBM and IBM processes. Ghosh et al. [67], reported that the materials through the hot stream can be mixed and new composite materials can be produced prior to deposition on substrates. This feature increases the flexibility of HVSPS coating process. On the other hand, there is no need for the deposited HA particles to perform a heat treatment as the particles have been already passed through a flame stream [68]. It has been reported by Forg et al. [69], that with increasing the velocity of the flame stream, the porosity of the coating layer increases while the velocity and porosity can be controlled in real-time. Based on research by Bernstein et al. [70], finer powders can be utilized in specifically designed fluid media in order as carriers. In this case, a finer coating structure with less unintentional porosity forms on substrates. Taking advantage of HVSPS characteristics in determining coating thickness, the gap between thin-film fabrication (chemical and physical vapor methods) and thick layers created by thermal spraying can be covered [71,72].

Although this process offers interesting advantages, the nature of HVSFS is a thermal coating method, and residual stresses will remain in the deposited structure. Norouzi et al. [74], suggested that with a decreased thickness of the deposited coating layer the chance of thermal cracking and coating failure decreases. They reported that the coating/substrate bonding is under the direct influence of the working parameters of HVSFS, i.e., flame stream velocity, oxygen/fuel ratio, and the distance between the nozzle and the target substrate. Gadow et al. [75], deposited bio-ceramic materials and reposted that with a change in the suspension composition, carrier liquids, and particle sizes, HA deposition takes place in a higher quality. They suggested that use of diethylene glycol (DEG) as an alternative to water-based media, significantly enhances coating adhesion to the substrate and offers a less porous microstructure. Chen et al. [76], reported that introducing metal oxide particles to the coating stream enhances the thermal stability of the substrate because the first layer of the deposited materials acts as a thermal barrier and protects the substrate against thermal exchange to the successive coating layers. In another study by Gadow et al. [73], they implemented metal oxide materials suspended in isopropanol to coat pure Ti substrate in order to form a protective layer. They reported an increase in the coating surface quality and a significantly finer microstructure than the one obtained by conventional thermal coating processes. However, they found slightly lower microhardness values of the top surface of the coating layer compared to those of the conventional thermal techniques. Table 1 presents their findings in brief.

**Figure 4:**
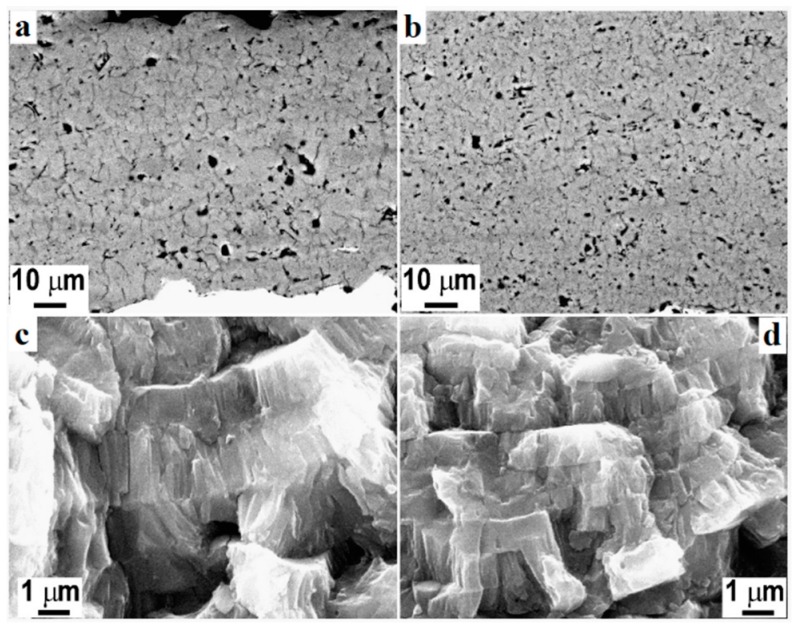
Polished cross-section of atmospheric plasma spray (APC) (**a**) and high-velocity oxy-fuel plasma coating (HVOF) (**b**) TiO_2_ coatings and respective fracture sections (**c**,**d**) [80].

Figure 4 represents an HVSFS-treated Ti substrate. HVSFS process can be optimized based on different working parameters. As with any other experimental method, these parameters can affect the performance of the process. It has been claimed that an increase in the rate of oxygen flow, increases the rate of coating/substrate adhesion [75]. The same trend has been observed regarding the simultaneous increase of oxygen and fuels rate [77]. With an increase in these two parameters, the temperature of the flame stream rises melting the involved particles inside the flame stream. Due to the acceleration of these particles, they may penetrate to the top layers of the substrate. At the same time, after partial melting, they become softer. Combining these two characteristics, they form a strong bonding to the substrate. However, excessive increase of the fuel and oxygen rate may increase the flame temperature to a point that decomposes the deposited materials and alter their functionality. The other affecting parameter is the distance of the nozzle head to the substrate. It has been reported that a shorter distance results in an increased bonding of the deposited materials to the substrate. However, with an inappropriate decrease in the distance, the formed turbulence may cause less deposition of materials due to splashing [78,79].

### 2.3. Plasma Spray Coating

**Figure 5:**
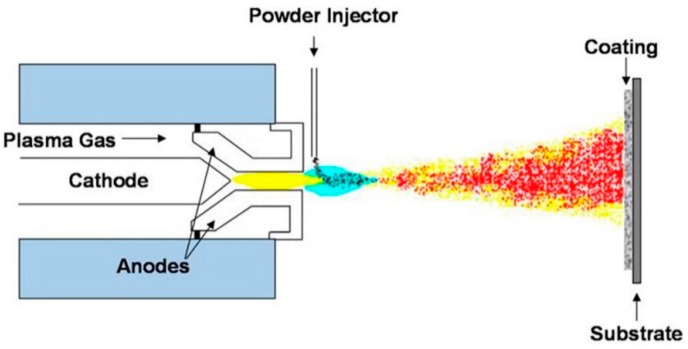
Schematic plasma spray coating technique and the targeted substrate [89].

One of the most investigated methods of material deposition is a plasma spray coating. Thanks to its ability to deposit metallic alloys, oxides, and ceramics, this process offers a wide range of applications in biomaterial and body implants coatings [81,82]. This process is similar to HVOF and HVSFS coating techniques. Figure 5 represents a schematic overview of a working plasma spray setup and the position of the targeted substrate to perform an effective HA coating experiment. A flow of solid particles is merged into a hot and accelerated plasma stream. Due to the high temperature of this process, there is a possibility of forming composite coating materials. The accelerated particles get hot and soft through the plasma stream and cover the surface upon impact to the substrate. The mechanism of coating is either by penetrating to the substrate or by deforming the particles due to the impact of energy. In the latter case, the flattened particles accumulate after a series of spraying and cover the surface of the substrate [83]. In addition, this process is capable of holding different atmospheric protections, i.e., air, inert gas, or vacuum. Narayanan et al. [84], reported that although the plasma jet reaches the high temperatures of 10,000 K or higher, a drastic temperature drop occurs after the plasma jet exits the nozzle tip. This process enjoys a high range of applications from biomedical implant coatings to fabricating thermal barrier coats. It has been reported that the plasma-treated surfaces present a high adhesion of coating/substrate [85]. Oehr et al. [86] coated a Ti plate with plasma spraying and deposited HA particles on the substrate. They observed an enhanced coating adhesion with improved osseointegration properties. Fatigue performance of titanium alloys is important regarding various applications [87], therefore, many studies were carried out to enhance the fatigue properties of these components. Yoshinari et al. [88] investigated the effect of HA coating thickness on the fatigue of Ti6Al4V alloy. They reported coating thicknesses of 25–100 μm do not show any negative effect, while an increase in thickness to 150 μm reduces the fatigue properties of the mechanical part. According to their results, increasing the thickness of the coating increases the difference between the material behavior of the substrate and the coating layer. Moreover, they discussed the excessive thermal shock, which is applied to the substrate during a thicker coating formation, causes micro-crack formation in both the coating layer and the substrate, reducing the fatigue life of the whole component.

**Figure 6:**
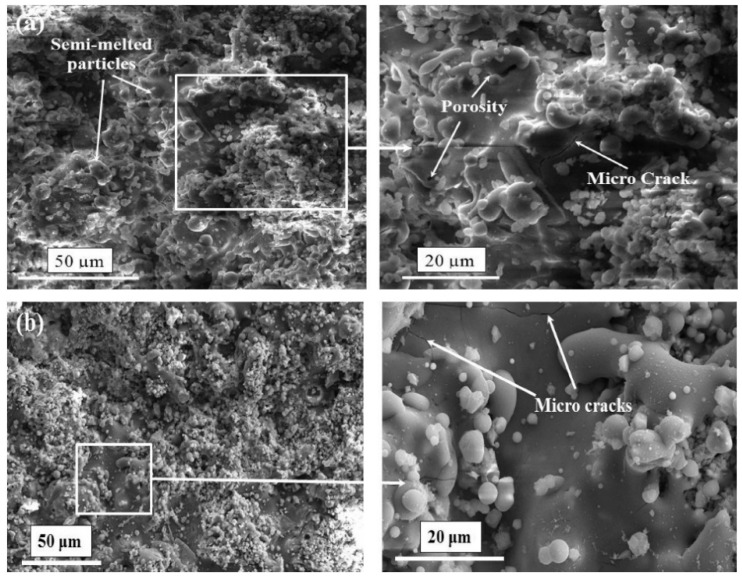
Morphology of HA-Zirconia coating stainless steel 316L substrate at different magnifications (**a**) as-sprayed (**b**) heat treated [99].

Similar to HVOF, a modification can be made to plasma spray coating to change the feedstock state. Gross et al. [90] introduced a suspension intake to the plasma jet and reached to thin-film coatings of 5–50 μm thickness. The process of utilizing suspension in the plasma spray coating is known as suspension plasma spray (SPS). The thickness of SPS-treated layer is significantly lower than the ones made by conventional plasma spray coating [91]. Different working parameters can affect the deposition quality. These parameters are particle size and feed rate of the feedstock, and atmosphere and acceleration of the plasma jet. Based on different combinations of the mentioned parameters, different deposition thicknesses ranging from sub-micron to 300 μm can be achieved [92,93]. For instance, Reitman et al. [94], discussed a decrease in coating/substrate adhesion in higher plasma jet temperatures due to higher amorphous HA content of the coating layer. Besides optimizing the working parameters, post-processing is a solution to improve the coating quality. By annealing the coated samples at 700 °C for 1 h, Lynn et al. [95] increased the coating purity and observed a crystalline structure of HA. Basu et al. [96] investigated the effect of elevated annealing temperature (up to 1100 °C) of HA-coated Ti substrate and claimed that annealing at higher temperatures, results in the formation of more Ca and Ti oxides in the coating. Zheng et al. [97] utilized the addition of Ti particles to HA particles in plasma spray coating and reported a significant enhancement of coating/substrate adhesion with increasing Ti content of the feedstock. Figure 6 represents a composite HA-Zirconia coating layer on a stainless-steel substrate. As can be seen, the heat treatment process seals a number of micro-pores, but there is no significant change in the appearance of micro-cracks. Singh et al. [98] modified the composition of the feedstock and proposed a new composition of 10 wt% (80Al_2_O_3_-20TiO_2_) on Ti6Al4V substrate and reported an enhanced bonding strength of 30 MP. Table 2 summarizes the properties of different plasma spray coatings.

### 2.4. Electrochemical Coating Techniques

**Figure 7:**
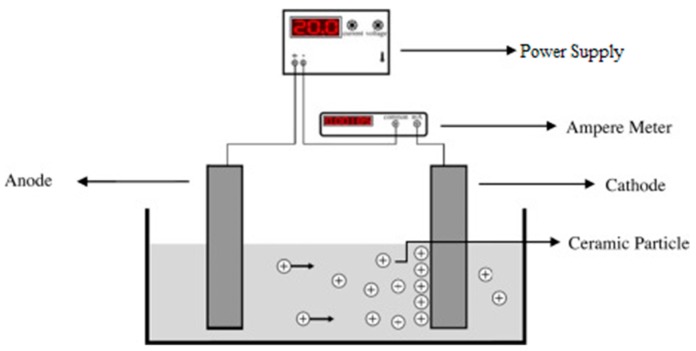
Schematic view of simple electrophoretic deposition process [108].

In the electrochemical coating technique, substrates undergo a series of electrochemical processes prior to being completely coated. Figure 7 represents a schematic overview of an electrophoretic coating setup. This process utilizes the potential difference between cathodic and anodic poles of an electrical circuit to form micro-arcs or to exchange ions between anion and cation sides [100]. The coating process takes place in two steps called electrophoretic and electrolytic deposition. The former is responsible for depositing large suspension particles existing inside the electrolyte, while the latter is in charge of depositing fine materials and structure. These two steps may be considered as two distinct processes or as separate steps of a single coating process [101,102]. In electrochemical processes, desired coating materials, e.g., CaP precursors, are dissolved in the working electrolyte. The highest number of applications of electrodeposition coatings done on Ti and Ti alloys substrates [103]. One of the characteristics of the electrochemical coatings is the uniformity in thickness of the deposited layer throughout the substrate [104]. Zhao et al. [105], investigated the applications of Pt and graphite anodic electrodes and reported an increased rate of deposition and enhanced coating quality. Although this process is considered a low-temperature method, the coated substrates have to undergo a series of densification and sintering in the furnace. The reason is that the surface of the coated substrate is not a compact structure due to the large suspension particle deposition during the electrophoretic step [106]. Due to electrochemical facts, the sharp edges, such as micro-cracks, are more prone to ion exchange, and as a result, they undergo a higher rate of material deposition. This characteristic guarantees the highest value of homogeneity and integrity in the coating layers [107].

**Figure 8:**
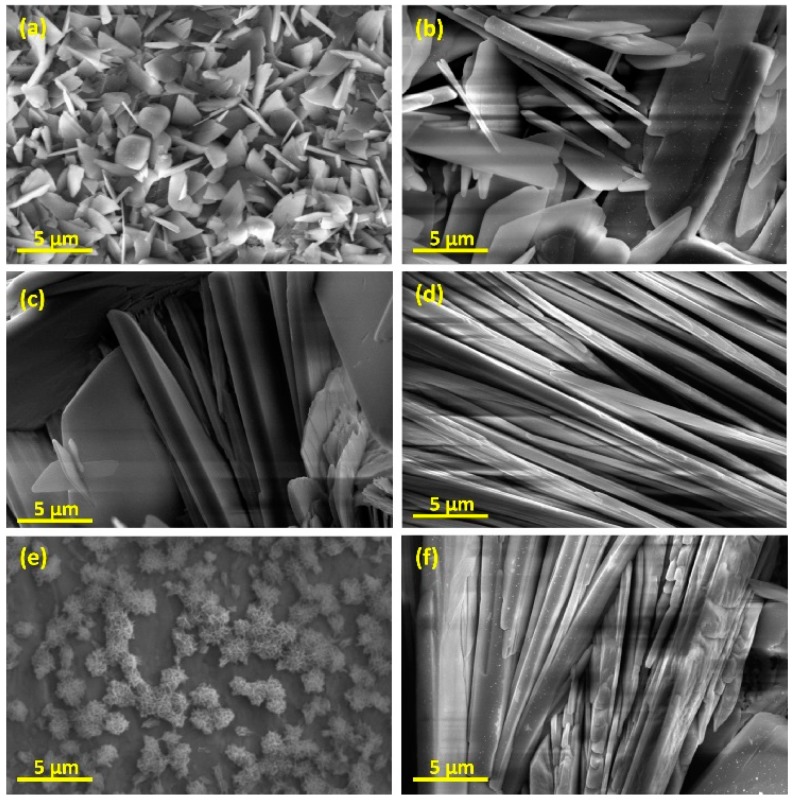
SEM micrographs of HA-coated samples by electrodeposition at (**a**) 1 V, (**b**) 2 V, (**c**) 3 V, (**d**,**e**) 5 mA/cm^2^ and (**f**) 10 mA/cm^2^ [122].

Peng et al. [109] investigated electrochemical coating of CoCrMo substrates and proved that although the thickness of the coating layer was decreased after sintering and annealing processes, the adhesion of HA deposited layer was significantly enhanced. The same results were reported by Zhang et al. [110] after annealing HA coated substrates at 500 °C for 1 h. However, they reported that due to the nature of electrochemical reactions in water-based electrolytes, a notable volume of hydrogen and air bubbles form on the surface of the cathode (the substrate) and prevent the complete HA deposition process. This discontinuity in the deposition step results in less uniformity and integrity of the coating layer. Moreover, after the sintering and annealing processes, these defects may get more intense due to shrinkage [111,112,113]. To solve this issue, it was recommended to implement H_2_O_2_ as a replacement for water. Because peroxide cancels the deteriorating effects of released H_2_ gas [114]. In addition, utilizing pulsed power supplies can result in enhanced coating quality and thickness while the uniformity is improved [115]. They suggested a higher off-time of the pulse to let the HA structures nucleate and grow easily. Xavier et al. [116] reported an increased apatite crystal formation after immersing the samples in SBF. There are different types of electrochemical coating processes known as micro-arc oxidation (MAO) and anodization. The voltage range, at which MAO occurs is comparably higher than that of anodization [117]. However, anodization is mostly considered as a pretreatment to electrochemical coating processes, whereas MAO is known as a separate coating technique [118,119,120]. He et al. [121], reported a successful deposition of a porous Al_2_O_3_ structure on Ti substrate utilizing anodization pretreatment. They observed an increase in CaP structure nucleation inside the pores of the coating layer. Figure 8 depicts the effect of different parameters on the morphology of the electrodeposited HA coating layer. In constant voltage mode, with an increase in coating voltage, the needle structure increases in size. While the process is run under the constant current mode, in the current density of 5 mA/cm^2^, the deposited material structure is similar to snowflakes while with increasing the current density to 10 mA/cm^2^, the materials deposition takes place in needle structures again.

According to many research reports, the addition of materials such as ZrO_2_, carbon nano-tubes (CNT), and titanium dioxide (TiO_2_) enhances HA coating performance [123,124]. In addition, it was observed that addition of other coating layers on the initial one (increasing the thickness by deposition of multilayered coating) increases the corrosion resistance and enhances ion release behavior of the coated substrate. This technique is called multi-walled coatings [125]. Henriques et al. [126] observed that a post heat treatment significantly improves the coating/substrate adhesion and the nucleation of CaP microstructure. Park et al. [127] reported an enhancement in corrosion resistance and stability of composite CNT/HA coating resulted in significantly increased biocompatibility. Yuan et al. [128] observed higher thickness homogeneity and enhanced coating/substrate adhesion in HA layers deposited on stainless steel. More information can be found in the literature [129,130]. Table 3 represents a brief comparison between different characteristics of the different methods of deposition.

## 3. Properties of Coating Layers

**Figure 9:**
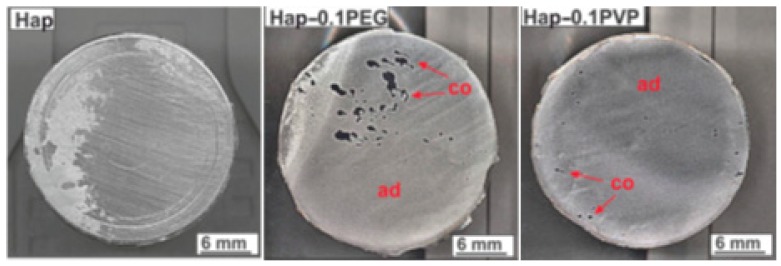
Surface of the coated samples and the detached coatings after adhesion strength tests [136].

Coating processes and the materials deposited on any substrate are assessed based on their performance. Some of these important criteria are the deposition-substrate bonding strength, the coating thickness, the corrosion resistance of the coating layers, and the stability of coatings in different working conditions. Bonding between deposited layers and substrates, called adhesion strength, is evaluated by applying a stress to the coating layer and measuring the highest strength value, at which no breakage or delamination occurs. The coating layers adhesion have to be equal or higher than the human bone stiffness [134]. To enhance the coating layer adhesion, several studies are carried out to investigate the chemical composition of the deposited materials. As an instance, Zhang et al. [135] reported up to 35% enhancement in adhesion of sol-gel deposited HA layers on Ti substrates with an increase in fluorine content of the coating medium. In addition, they claimed that with an increase in the heat treatment temperature, the adhesion increases, as well. Sopcak et al. [136] utilized the addition of polyethylene glycol (PEG) and polyvinylpyrrolidone (PVP) polymers into HA containing coating medium and coated Ti samples with electrostatic spraying. In a comparison between substrates coated using different process parameters, they observed a significant increase of the coating adhesion strength with an increase in PEG and PVP compounds (up to 400% of the sole HA-coated samples). Figure 9 represents the surface conditions of different coated samples by HA, PEG, and PVP. In an investigation by Rocha et al. [71], Ti6Al4V substrate was coated by thermal spraying, and HA-TiO_2_ ceramic was deposited on the surface. They observed the adhesion strength of 30 ± 2 MPa, which was higher than what was reported in the literature. Fujihara et al. [137] compared thermal spraying-coated samples with ion/laser beam-treated samples and reported that after long-term in vivo tests, 90% of the thick coatings of the first group failed, while ion/laser beam-deposited HA provided higher durability despite their considerably lower thickness.

**Figure 10:**
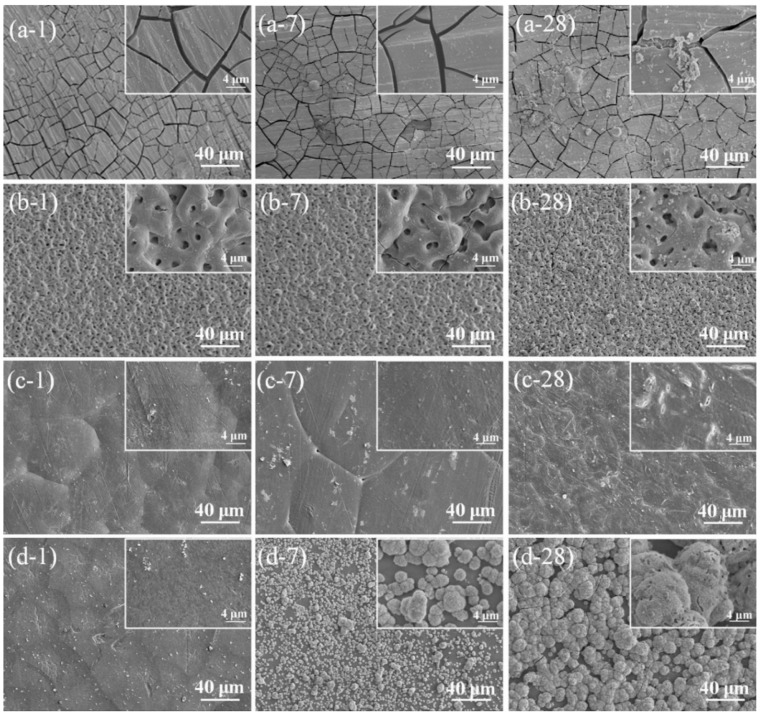
Degraded Mg-1.2Zn-0.5Ca alloy coupons aged at different durations after in vitro immersion in the SBF solution for different times of 1, 7, and 28 days. (**a**) Degradation of Mg-1.2Zn-0.5Ca (**b**) degradation of PEO coated Mg-1.2Zn-0.5Ca samples, (**c**) degradation of PEO-coated Mg-1.2Zn-0.5Ca samples sealed with PCL, and (**d**) degradation of PEO/PCL-coated Mg-1.2Zn-0.5Ca samples dipped in PDAM. In the non-coated samples, a severe cracking scheme is visible which is a result of dehydration of Mg(OH)_2_ as the main byproduct of corrosion. PEO-treated samples represent no severe sign of corrosion after 28 days. The PEO/PCL-treated samples provide higher corrosion resistance with a slight degradation of PCL layers on the PEO-deposited layer. Finally, the PEO/PCL/PDAM samples provide high corrosion resistance with no signs of pitting or cracking on the surface while sites of HA formation can be seen obviously on the outer layer which is resulted by addition of PDAM [149].

Another factor affecting the protective properties of coating layers is the layer thickness. It has been reported that hot-isostatic press can form thick coatings of up to 200 µm that is close to the range of thermal spray coating thicknesses. High-temperature processes such as ion/laser beam material deposition provide the least thickness of coatings, while they have a reliable adhesion and protection. In addition, low-temperature processes such as electrochemical processes and sol-gel have variable thicknesses and offer multi-layered coating layers. Although the last two types of the material deposition processes are very flexible in coating composition and thickness, they cannot offer a very thick coating layer, and they are categorized as moderate thicknesses coating techniques [138,139,140,141]. Based on the previous discussion, it has been revealed that the protective purposes of ion/laser beam coating layers have maintained their functionality during long-term applications offering the best performance. However, the disadvantages of these two processes are the high costs of the process and equipment and their inability to coat complex geometries [84]. Based on the coating layer adhesion and thickness, it is important to have a higher physical and chemical stability of the deposited materials. Stability of a coating means the interaction of the deposited materials under different chemical and mechanical conditions while the coated implants are in use. Degradation of the coating material in physiological fluids can be an example of these conditions [142,143]. Figure 10 depicts the degradation of body implants in different conditions after a specific number of days of application. The samples are coated by plasma electrolytic oxidation (PEO), then sealed with polycaprolactone (PCL), and finally dipped in polydopamine (PDAM). The final sample was then washed to remove loosely bonded PDAM from the coating surface. In many applications, the implants are needed to degrade in the human body after a specific time of utilization, e.g., after a fractured bone is healed, but it is important to maintain the minimum function time [144,145]. Mg implants are very vulnerable to the body fluids, and they corrode quickly after exposure. However, these materials are good candidates for implants due to their biocompatibility properties. Many researchers have tried to coat Mg substrates in an engineered way to provide enough time for the implant to serve inside the human body and to dissolve gradually and disappear after the healing period. However, if Mg samples are coated with durable coatings, such as the ones provided by ion beam deposition, the implants remain inside the patient body for a long time, and another surgery is needed to remove them [146,147,148]. This example highlights the importance of stability of the coating materials.

**Figure 11:**
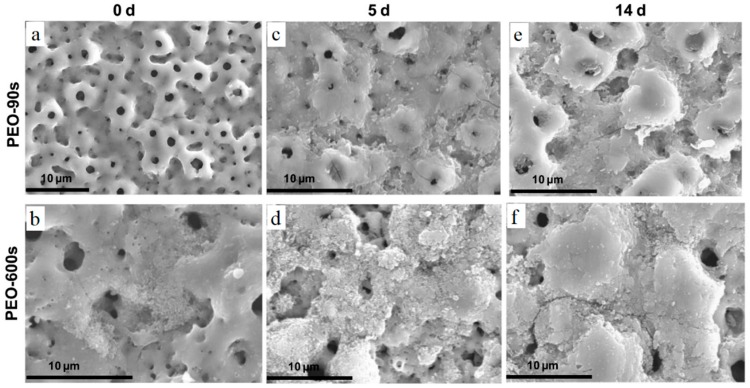
SEM micrographs of MAO-treated Ti CP before and after immersion in α-MEM without cells: (**a**) MAO-90s, (**b**) MAO-600s, (**c**) MAO-90s 5-day immersion, (**d**) MAO-600s 5-day immersion, (**e**) MAO-90s 14-day immersion, (**f**) MAO-600s 14-day immersion [158].

One of the most important applications of the coating layers is to increase the corrosion resistance of a substrate, which is exposed to a harsh environment. This means that not all of the bio-applications need a coating layer, which degrades gradually and disappears after a period of time [150]. The corrosion resistance of the coating layers is desired for two reasons: (1) The protection integrity of the implants and (2) to prevent ion release into the body fluids. Metallic ions are the products of corrosion of metallic implants and can be harmful to human health, especially when they are higher than a specific dosage. A significant instance of these materials can be the released Ni ions from NiTi body implants [4]. Based on the literature, although CaP-based coatings enhance the bone ingrowth and form a quick and strong interface between bones and implants, they suffer from low corrosion resistance, compared to metallic oxide coatings, such as TiO_2_, Al_2_O_3_, etc. As a result, solid implants are exposed to corrosive media and lose their mechanical properties that can be considered as a malfunction in healing a fractured bone [151]. However, various techniques, such as surface finish, surface blast, heat treatment, change in composition, etc., have been proposed to modify the surface quality and the deposited materials structure, thus to enhance the corrosion resistance of CaP-based coatings [152,153]. Zhao et al. [154] have utilized PCL to seal porous structure of HA coating. They obtained HA/PCL composite scaffolds with higher corrosion resistance. Based on the surface finish and porosity of the deposited layer, many considerations can be considered to improve the corrosion properties of a coating layer. Heat treatment is another method of coating modification that improves the crystallization and enhances corrosion resistance. Xia et al. [155] investigated an HA-coated Ti6Al4V substrate by the thermal spraying technique with a successive hydrothermal treatment (HT) resulted in an increased crystallinity. They reported an enhancement in coating degradation after the HT process. Moskalewicz et al. [156] implemented TiO_2_ nanoparticles to enhance the electrochemical corrosion of Ca/P-based coatings. They introduced a specific ratio of TiO_2_ nanoparticles to HA solutions prior to implementing the coating process. Corrosion testing of the coated substrates revealed an enhancement in the corrosion resistance and bioactivity of the deposited layer. In another study, Ionita et al. [157] achieved a TiO_2_/HA coating layer with enhanced corrosion properties and bioactivity, while the deposited materials presented active antibacterial properties. Santos-Coquillat et al. [158] modified Ti substrate by utilizing MAO. They observed an increased corrosion resistance after depositing Ca/P structures on the substrates. In addition, they implemented cell culture experiments and reported a good cell adhesion with no or lower signs of toxicity after MAO surface treatment. Figure 11 represents the MAO-treated surface. The difference in coating structure results from different exposure time of specimens. In the two groups, the microstructure is porous with different pore sizes reflecting the voltage of current density applied to the sample surface. After exposure of the coated parts to the cell platform, it can be seen that in both cases, the cells are growing well, which reveals the suitability of the coating composition and microstructure.

Overall, various parameters are effective in obtaining a reliable coating layer, which is suitable for bio-purposes. Although at first glance, some of these parameters look more important than the others, they are all in a direct interrelationship and ignoring one may affect the overall performance of the coated surface. In most of the bio-applications, corrosion resistance, stability, composition, and bioactivity of the coating layers are simultaneously in action to offer the best protection and healing properties.

## 4. Summary

Many metallic and non-metallic materials are used as body implants to facilitate patient healing. However, to date, the ratio of implementation of non-metallic to metallic implants is negligible. As a result, a deep investigation is needed to select the best metallic compound or alloy to derive the best performance for implants. Unfortunately, metallic implants suffer from different deficiencies, such as low/high corrosion resistance, releasing toxic ions, and low biocompatibility. To resolve this issue, many surface modification methods are proposed that coating techniques are among the most important ones. Each of these techniques demonstrates different capabilities and one has to select a coating method based on their needs and applications. Among these techniques the most common ones are sol-gel, high-velocity suspension spray, plasma spraying, and electrochemical coating processes. Table 4 represents a summary of the materials deposited on different substrates using sol-gel, HVOFS, plasma spraying, and electrochemical processes.

I. Sol-gel is the cheapest method that has high flexibility in the composition of the deposited materials and can form deposition on complex substrates.
II. Electrochemical processes are in the same run while they are applicable only to conductive materials. This limits their applications on surfaces that are not electrically conductive.
III. High-velocity flame and plasma spraying processes in different forms can produce considerably thick depositions. However, these processes implement a high-temperature thermal process to melt the feedstock or change them into a semi-solid form. A high thermal gradient may affect the properties of a metallic or ceramic substrate while makes these processes unable to coat polymeric and plastic substrates.

Therefore, a method should be selected based on the desired needs and functionalities. Many scientific reports have been revealed that multi-layered coatings are achieved to employ the advantages of different deposition processes while diminishing their drawbacks. Although current deposition techniques are reliable means of surface protection, there is still a need for finding better solutions by introducing new techniques and materials for coating. Perspective studies can focus on developing combined processes with fewer side effects, higher control on deposition rate, less cost and higher ease of use.

## Figures and Tables

**Table 1:** Summary of characteristics of different materials used for coating of pure Ti with HVSFS process [73].

Spray Material	Microhardness (HV)	Porosity (%)	Roughness (Ra, µm)	Phase Composition
Al_2_O_3_	620–880	5	0.58	Mainly γ
TiO_2_	1000	0–0.05	0.65	Mainly anatase
Cr_2_O_3_ (in propane)	1400–1800	4–5	0.47	Hexagonal
Cr_2_O_3_ (in ethane)	1100–2000	7–10	0.48	Hexagonal
3YSZ	715–816	<1	1.75	Tetragonal

**Table 2:** Comparison between characteristics of different modes of plasma spray coating.

Process	Speed	Quality	Cost	Thickness
Air plasma	High	Low	Low	High
Inert gas plasma	High	Moderate	Moderate	High
Vacuum plasma	Low	High	High	High
Suspension plasma	Moderate	Moderate	Moderate	Low

**Table 3:** A qualitative comparison of properties of different deposition processes.

Method	Nature of Coating	Thickness	Porosity	Adhesion	Flexibility	Speed	Cost	References
Sol-gel	Physical	Low-medium	Medium-high	Medium-high	Very high	Very low	Low	[35]
HVOF	Thermal	High	High	High	Moderate	high	Moderate	[131]
Plasma spray	Thermal	High	High	High	High	High	Moderate-high	[132]
Electrochemical	Chemical	Low-medium	Low-medium	High	Very high	Moderate	Low	[133]

**Table 4:** Summary of materials deposited on different substrates via various coating techniques.

Process	Deposited Materials	Substrates	Reference
Sol-gel	HA, TiO_2_, PCL	Ti alloys, ceramic, Stainless steel	[35,37,59,60]
HVOFS	Metal oxides such as TiO_2_, Al_2_O_3_, Cr_2_O_3_, ceramics	Ti alloys, Stainless steel, ceramics	[77,78,79,80]
Plasma spray	HA, HA-Zr_2_O_3_, Al_2_O_3_-TiO_2_	Ti alloys, Zr alloys, ceramics, metallic alloys	[85,86,87,88,89,90]
electrochemical	ZrO_2_, TiO_2_, MgO, CNT, HA, HA-TiO_2_, SWCNT, MWCNT	Metallic alloys, ceramics, Ti alloys	[119,120,121,122,123,124,125]
